# Census Tract Demographics Associated with Libraries’ Social, Economic, and Health-Related Programming

**DOI:** 10.3390/ijerph19116598

**Published:** 2022-05-28

**Authors:** Sasha A. Fleary, Carolina Gonçalves, Patrece L. Joseph, Dwayne M. Baker

**Affiliations:** 1 Community Health and Social Sciences, CUNY Graduate School of Public Health and Health Policy, New York, NY 10027, USA; 2 Eliot-Pearson Department of Child Study and Human Development, Tufts University, Medford, MA 02155, USA; carolina.goncalves@tufts.edu; 3 Gillings School of Global Public Health, University of North Carolina, Chapel Hill, NC 27514, USA; patrece.joseph@unc.edu; 4 Urban Studies Department, CUNY Queens College, Queens, NY 11367, USA; dwayne.baker@qc.cuny.edu

**Keywords:** public libraries, public health, community programming, social determinants of health, health inequities, health disparities, underserved communities, census

## Abstract

**Background**: Public libraries can contribute to reducing economic, social, and health inequities through their programming and practices. However, the extent to which libraries regularly provide programming that improve the social determinants of health (SDH) in underserved communities is unclear. **Objective**: This study explored the relationship between census tract demographic characteristics and library programming implicated in the SDH for underserved groups at risk for health disparities. **Method:** A stratified random sample of libraries (*n* = 235) who completed the 2017 Public Libraries Survey were recruited. Librarians completed surveys about their libraries’ economic, social, and health-related programming. Libraries’ census tract demographic characteristics were taken from the 2013–2017 American Community Survey. Linear regressions were estimated to determine the relationship between relevant census tract demographic characteristics and programming offered at libraries in the census tracts. **Results:** Higher proportions of racial and ethnic minorities were associated with more frequent economic and social programs, but results were mixed for health-related programs. Lower proportions of populations with no more than a high school diploma or GED were related to more frequent economic, social, and health-related programs. **Conclusions:** The inequitable distribution of SDH-related library programming highlights gaps in libraries’ responsiveness to community needs. Libraries’ programming likely perpetuate systemic inequities.

## 1. Introduction

According to the American Library Association (ALA), there were an estimated 116,867 libraries in the United States in 2019 and approximately 9057 of them were public library branches [1]. A 2016 Pew survey found that 53% of Americans 16 years and older had some interaction with public libraries in the last year [2]. The public library is viewed as a safe gathering space that promotes community, a source of trusted information, and a place to help individuals find health information and job opportunities [3,4,5]. The public library also aims to promote civic education, bridge communities, and provide a forum for public dialogues through programming such as English language learning classes, resume writing consultations, and several other workshops [3]. Morgan et al. [6] found that libraries were disproportionately used by vulnerable populations including individuals experiencing mental illnesses, homelessness, and those recently migrated.

Public libraries emerged throughout the United States after the Revolutionary War to fulfil the need for spaces where people could access and discuss literature [3,7]. Later, libraries offered opportunities for women who were banned from formal learning spaces and eventually became one of the very first public spaces where Black people were allowed to frequent despite Jim Crow laws [7]. Over the centuries, libraries have evolved into a quintessential part of how many Americans learn and engage with the community. In response to an influx of immigrants arriving to the United States from 1880 through 1920, public libraries amassed foreign-language collections that reflected the particular needs of the ethnic and national groups represented in the libraries’ respective communities and provided programming for these immigrants to learn to speak, read, and write English [7]. Therefore, libraries are intended to be a welcoming space for all people and have a history of directly and indirectly addressing issues related to racism, sexism, and other societal problems through their programming and practices. However, like many US systems, some of the public library’s programming and practices may perpetuate systemic inequities that continue to marginalize and stagnate underserved communities.

Systemic inequities rooted in marginalization, racism, and discrimination account for poor health [8,9], social and economic immobility [9,10], and lower quality of life [11] for underserved groups. Improving the wellbeing of impacted communities requires changes to how systems function as well as improved and equitable access to resources. As the role of the public library evolves, it is important to consider how this trusted system may serve to improve the conditions in which people live, work, grow, and learn [12,13], that is, their social determinants of health. Libraries may play a pivotal role in reducing inequities through their programming, especially if they are responsive to the needs of their patrons. For example, libraries in communities with high rates of recently incarcerated or currently incarcerated individuals may provide re-integration and/or family support programming [14]. Such programs have the potential to reduce recidivism and improve economic mobility, and family mental health and well-being—all of which impact long-term health outcomes and quality of life. In other examples, staff at libraries spend a lot of time providing assistance to individuals from vulnerable populations in addition to offering multiple social determinants of health-related programming [5,6].

Potential recipients of these programs and services are open to health-related library programming. Findings from Butler [15] highlighted that participants were willing to receive active education and support with health-related issues from libraries and these participants believed this could be accomplished through partnerships between libraries and health services. Lenstra [16] found the libraries’ movement-based programming (e.g., yoga, early literacy) brought new users to the library. In another study, Lenstra et al. [17] found that in addition to improving strength and mental health, strength-training programming at the library provided opportunities for older adults to socialize and learn more about what the libraries had to offer. A scoping review by Philbin et al. [18] mapped out the ways in which public libraries may advance population health through a range of services and programs that address critical social determinants of health. Results from this review concluded that libraries may promote access to healthcare through provision of direct healthcare services, health information, and linkage to services. Racial and ethnic minorities, individuals with low income, and those with low education have poorer outcomes related to their social determinants of health [12], economic immobility [9], lower quality of life [11], and poorer health outcomes [8,9]. Given this, libraries serving higher proportions of individuals with these demographic characteristics should offer frequent programming that provides access to resources and information to improve their patrons’ financial prospects, health, and other social determinants of health.

## 2. Objective

The extent to which libraries regularly provide programming that improve social determinants of health in communities most affected by systemic inequities is unclear. Therefore, the purpose of this study was to explore the relationship between census tract demographic characteristics and library programming that have the potential to improve social determinants of health for groups who are underserved and at risk for health disparities.

## 3. Method

### Procedures

The Tufts University Social, Behavioral and Educational Research Institutional Review Board deemed this study as exempt. Librarians from public libraries across the United States were recruited to complete a short survey about the programming offered at their library. Data from the 2017 Public Libraries Survey (*n* = 17,452 library entries) were used to randomly recruit libraries for participation in our study. First data were cleaned to exclude libraries that were open less than 48 weeks per year, only included book mobiles, and were closed or temporarily closed (*n* = 1448 libraries removed). The libraries for each state were sorted into separate files by state and locale. Locales represented the size of the community in which the library was located and its proximity to urban and metropolitan areas. There were four locales: cities, suburbs, towns, and rural areas. All libraries were assigned a unique number (*n* = 16,004 libraries) and a random number generator was used to randomly select 10 libraries from each locale within each state.

**Figure 1:**
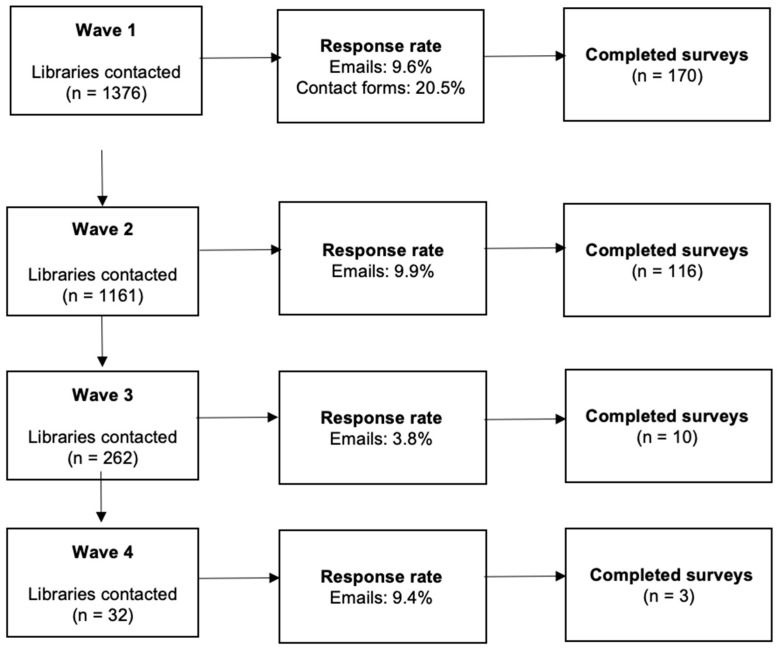
There were 64 libraries for which surveys were completed but zip codes were not available to link the library to its census tract.

To sample a representative list of libraries, there were four waves of recruitment: Waves 1 (initial sampling of libraries; September 2019), Wave 2 (resampling to recruit additional participants; March–April 2020), Wave 3 (targeted sampling to ensure representativeness across states and locales; May–June 2020), and Wave 4 (sampling of libraries in major cities; July 2020). After a random number generator was used to generate a list of 10 randomly selected libraries within each locale category per state, members of the research team verified that each of the randomly selected libraries was not an academic or government library and had a working website and available contact information including either an email address or contact form on their website. Libraries not meeting these criteria were excluded and in cases where there were less than 8 libraries, additional libraries were sampled by randomly generating additional numbers. Email addresses for the library directors were placed in an excel document. In cases where the director’s email address was not available, the general library email was used instead. Library branches and systems were contacted to complete the survey. In each wave, each library was emailed an individualized link to the survey. If the survey was not completed within a week, a reminder email that included the survey link was sent. Libraries that did not respond to the second attempt within a week of the reminder email were replaced with new libraries, which were sampled in a subsequent wave. Due to the very low response rate (see Figure 1), Wave 2 recruitment was launched and followed the same procedures as Wave 1 except only libraries with email addresses were retained. We opted not to use contact forms due to several issues (e.g., character limits, feedback from librarians discouraging the use of contact forms for this purpose). After Wave 2 recruitment, the sample was reviewed to determine the representativeness across locale groups and states. Wave 3 was initiated to recruit libraries in the states across the locale groups for which we did not receive a response. In Wave 4, only libraries from major cities were recruited. For Waves 3 and 4, the same procedures for sampling and contacting libraries for Wave 2 were followed unless all eligible libraries had been contacted in earlier waves.

During each wave of recruitment, libraries were sent an email that included information about the study and a survey link via Qualtrics. For Wave 1 only, libraries with online contact forms were placed on a separate list and the study information was entered into the contact form on the libraries’ website. A library director or someone knowledgeable about the libraries’ programming was asked to complete the survey. The survey took about 20 minutes to complete, and participants were offered a $10 Amazon electronic gift card. Librarians were also asked to indicate whether they would be interested in being contacted to participate in a follow-up individual interview.

## 4. Measures

### 4.1. Demographic Characteristics

Demographic characteristics were derived from the 2013–2017 American Community Survey 5-year estimates as provided by the National Historical Geographic Information System (NHGIS) [19] at the census tract level. These characteristics include proportions of residents with less than or equal to a high school diploma/GED, proportions of non-Hispanic Black, Native American and Alaska Native, Asian, or Native Hawaiian and other Pacific Islander residents, Hispanic/Latinx residents, unemployed residents, and residents in poverty.

### 4.2. Libraries Programming

Library representatives were asked to indicate whether their libraries offered programming implicated in economic mobility (i.e., financial literacy, job preparedness, starting a small business, college preparation), health (i.e., nutrition, physical activity, chronic illnesses, mental health), and other social determinants of health (i.e., health insurance, housing, immigrant rights, English language learner education). For libraries that offered programs, representatives indicated the frequency with which the programs were offered. Response options included less frequent than once per year (1), once per year (2), a few times per year (3), a few times per month (4), a few times per week (5), and daily (6). The two questions were combined into a single variable with responses of no on the former question coded as 0 (not offered).

### 4.3. Statistical Analyses

All analyses were conducted in SPSS. Bivariate and multivariate linear regressions were estimated to determine the relationship between relevant census tract demographic characteristics and programming offered at the libraries in the census tract.

## 5. Results

The final sample included 235 libraries and descriptive statistics are presented in Table 1 and Table 2. On average, 5% of adults 25-years and older had an education level that was less than or equal to a high school diploma/GED across census tracts of libraries surveyed. Regarding race, across the census tracts of libraries surveyed, on average, 11% were non-Hispanic Black, 2% non-Hispanic Native American/Alaskan Native, 3% non-Hispanic Asian, and 0.2% non-Hispanic Native Hawaiian/Pacific Islander adults. Approximately, 11% of adults in census tracts of libraries surveyed were Hispanic/Latinx adults. Across census tracts of libraries surveyed, an average of 7% of adults were unemployed and 16% experienced poverty.

When programs were offered, most libraries frequently offered programs a few times per month. The programs offered most frequently by libraries surveyed included financial literacy (54.5% of libraries), job preparedness (57%), and physical activity education (45.5%). Nutrition and/or healthy eating (43.5%) was offered daily at ~36% of the libraries. Programs or workshops offered less by libraries included small business ownership (30.6%), college preparation (34%), signing up for and/or understanding health insurance (37.4%), home ownership (26.8%), specific chronic illnesses (30.6%), and mental health (31.5%). The least frequently offered programs were English language learning programs (24.7%) and programs on immigration rights and issues (16.2%). When offered, English language learning programs were most frequently offered once per year.

### 5.1. Economic Mobility Programming

Bivariate and multivariate results are presented in Table 3. In bivariate analyses, higher proportions of non-Hispanic Asian populations were related to higher frequency of financial literacy programs offered (*β* = 0.14, *p* = 0.03). However, this statistical significance was not maintained in the multivariate model (*β* = 0.13, *p* = 0.09). Regarding job preparedness programs, higher proportions of non-Hispanic Black populations were associated with higher frequencies of job preparedness programs offered in bivariate regressions (*β* = 0.17, *p* = 0.01). In multivariate regressions, higher proportions of non-Hispanic Black (*β* = 0.23, *p* = 0.003) and Hispanic/Latinx (*β* = 0.16, *p* = 0.028) populations were related to higher frequencies of job preparedness programs offered. In bivariate analyses, lower proportions of individuals whose highest education was less than or equal to a high school diploma/GED (*β* = −0.17, *p* = 0.009), and higher proportion of non-Hispanic Black (*β* = 0.13, *p* = 0.047) and Asian (*β* = 0.17, *p* = 0.009) populations were related to higher frequencies of small business programming. These findings were consistent in the multivariate analyses for education (*β* = −0.28, *p* = 0.001) and non-Hispanic Black (*β* = 0.20, *p* = 0.008), but not for non-Hispanic Asian (*β* = 0.06, *p* = 0.43). In both bivariate and multivariate analyses, lower proportions of individuals whose highest education was less than or equal to a high school diploma/GED (*β* = −0.26, *p* < 0.001; *β* = −0.22, *p* = 0.005, respectively) and higher proportions of non-Hispanic Asian (*β* = 0.36, *p* < 0.001; *β* = 0.27, *p* < 0.001, respectively) and Hispanic/Latinx (*β* = 0.16, *p* = 012; *β* = 0.19, *p* = 0.004, respectively) in a census tract were associated with higher frequencies of college preparation program offerings. Noteworthy, the proportion of the population unemployed or experiencing poverty in census tracts were unrelated to any of the economic mobility programs offered at the libraries.

### 5.2. Social Programming

Bivariate and multivariate results are presented in Table 4. In multivariate analyses, higher proportions of non-Hispanic Native Hawaiian or Other Pacific Islander (*β* = 0.14, *p* = 0.047) and lower proportions of Native American or Alaska Native (*β* = −0.15, *p* = 0.034) populations and people experiencing poverty (*β* = −0.22, *p* = 0.009) within census tracts were associated with higher frequencies of health insurance programming. Regarding housing, in bivariate analyses, higher proportions of non-Hispanic Asian (*β* = 0.13, *p* = 0.046) and Native Hawaiian and Pacific Islander (*β* = 0.16, *p* = 0.017) populations and lower proportions of individuals whose highest education was less than or equal to a high school diploma/GED (*β* = −0.17, *p* = 0.011) in a census tract were related to higher frequencies of housing programming offered. In multivariate analyses higher proportions of non-Hispanic Black (*β* = 0.16, *p* = 0.036) and Native Hawaiian and Pacific Islander (*β* = 0.19, *p* = 0.008) populations and lower proportions of individuals whose highest education was less than or equal to a high school diploma/GED (*β* = −0.26, *p* = 0.002) were associated with higher frequencies of housing programming offered. In bivariate analyses, higher proportions of non-Hispanic Asian (*β* = 0.13, *p* = 0.041) and Hispanic/Latinx (*β* = 0.15, *p* = 0.026) populations were associated with higher frequencies of English language learning programming offered. However, in multivariate analyses, lower proportions of individuals whose highest education was less than or equal to a high school diploma/GED (*β* = −0.18, *p* = 0.030) and higher proportions of Hispanic/Latinx populations (*β* = 0.15, *p* = 0.029) were associated with English language learning programming offered. Regarding immigration rights programming, in bivariate analyses, lower proportions of individuals whose highest education was less than or equal to a high school diploma/GED (*β* = −0.16, *p* = 0.014) and higher proportions of non-Hispanic Asian (*β* = 0.24, *p* < 0.001) and Hispanic/Latinx (*β* = 0.21, *p* = 0.002) populations were associated with more frequent programming. In multivariate analyses, education (*β* = −0.27, *p* = 0.001) and Hispanic/Latinx (*β* = 0.23, *p* = 0.001) remained significant and non-Hispanic Asian (*β* = 0.11, *p* = 0.118) was no longer associated with immigration rights programming offered.

### 5.3. Health-Related Programming

Results are presented in Table 5. In bivariate and multivariate analyses, lower proportions of non-Hispanic Asian populations in census tracts (*β* = −0.12, *p* = 0.048; *β* = −0.15, *p* = 0.04, respectively) were related to more nutrition programs offered. In multivariate analyses, lower proportions of Native Hawaiian or Other Pacific Islander populations (*β* = −0.16, *p* = 0.023) were related to higher frequencies of physical activity programming offered. Regarding chronic illnesses, higher proportions of individuals whose highest education was less than or equal to a high school diploma/GED (*β* = −0.14, *p* = 0.028) was associated with less frequent programming. In addition to education (*β* = −0.18, *p* = 0.039), higher proportions of non-Hispanic Black populations (*β* = 0.17, *p* = 0.026) were associated with higher frequencies of chronic illnesses programming offered in multivariate analyses. For mental health, bivariate and multivariate analyses both implicate higher proportions of individuals whose highest education was less than or equal to a high school diploma/GED (*β* = −0.22, *p* = 0.001; *β* = −0.29, *p* = 0.001, respectively) in lower frequencies of mental health programming offered. Similar to economic mobility programming, unemployment and poverty status were unrelated to programs offered.

## 6. Discussion 

Racial and ethnic minorities, and individuals with lower education and lower income are disproportionately impacted by systemic inequities and are at higher risk for poorer outcomes associated with social determinants of health. Through their programming, libraries have the potential to reduce economic, social, and health inequities in their communities. This study found that social determinants of health-related programming offered by the libraries was related to the proportion of the population in the libraries’ census tracts whose highest education was less than or equal to a high school diploma/GED, were racial minorities, Hispanic/Latinx, unemployed, and below the federal poverty level.

Interestingly, proportions of populations with low education were unrelated or negatively related to economic mobility, social, and health-related programs offered. Given the relationship between education and economic mobility [20], health [21], and other social determinants of health [11], these results highlight how libraries may perpetuate systemic inequities. For example, fewer programs in census tract A where most individuals whose highest education was less than or equal to a high school diploma/GED compared to census tract B, will mean that a higher resourced group is given even more resources for success while a low resourced group stays stagnant. These discrepancies in programming widen the disparities and knowledge gap. Therefore, it is imperative that libraries examine and design their programming with consideration of their communities’ needs with the goal of empowering their community members to improve their life circumstances. To effectively do this, libraries need to focus on community members who do not regularly use the libraries as well as their committed patrons. 

Relationships between race and ethnicity and programming varied greatly. Overall, libraries in census tracts with higher proportions of non-Hispanic Black, non-Hispanic Asian, and Hispanic/Latinx populations were more likely to offer economic mobility and social programming. Libraries in census tracts with higher proportions of non-Hispanic Native Hawaiian or Other Pacific Islander were more likely to offer social programming and less likely to offer health programming. Health programming was also less common in libraries with higher proportions of non-Hispanic Asian populations. The extent to which the availability of these programs fully captures the needs of the specific racial and ethnic groups should be investigated further. For example, though English language learning and immigration rights programming were associated with communities assumed to have individuals who may benefit the most from them, it is possible that other racial minority groups may have as much of a need [22]. Further, given the disproportionate impact of obesity [23,24] and other chronic diseases [25,26] as well as the higher rates of mental health stigma [27,28] and lower mental health help-seeking [29] among racial and ethnic minorities, we would expect that as racial and ethnic minority populations increase, health-related programming offered at the libraries in their census tract would also increase.

Most surprisingly, the two direct indicators of low income, poverty, and unemployment, were unrelated to all libraries programming except for health insurance workshops where poverty status was negatively related to programming. According to PEW, 41% of low-income individuals and 30% of individuals with less than a high school diploma visited the library in 2015 [2]. These individuals visit the library to use the internet, computer workstations, get help from a librarian, or to sit, read, or study [2]. Research by PEW also suggests that many individuals believe that libraries should provide pathways to economic opportunity such as business development, jobs search, and workforce skills [2]. Given that all these individuals would greatly benefit from the programs studied and these programs may serve to move them out of their current social and economic standing, it is important to address the barriers that prevent libraries from providing these programs and reimagine the use of libraries for effectively building marketable work skills in underserved communities.

Individuals with lower education may have multiple skills and talents that may be monetized but lack the cultural capital, information, and resources to do so. Free small business programming may allow for information dissemination and skills building necessary for individuals with lower education to consider and be more successful at entrepreneurship [30]. Similarly, individuals may be stagnated in their educational growth due to lack of information on how to enroll in and fund their college education and alternatives to college education. Further, these adults may have children who are interested in attending college but may not do so or incur unnecessary debt because parents lack the information to guide them. College preparation courses, especially courses that involve parents may play a critical role in fostering intergenerational educational attainment and, by extension, economic mobility [31].

The inequities associated with low education and low income are often compounded for individuals who belong to extremely vulnerable groups including those without adequate housing, English language learners, immigrants, and those with chronic and mental illnesses [32]. These groups navigate complicated systems and may therefore be in dire need of resources and guidance for successfully navigating these historically racist and discriminatory spaces [33]. Language barriers may hinder economic mobility, self-advocacy, and health outcomes and these barriers may be most pronounced for individuals with low education. Therefore, fewer English language learner programs in census tracts with lower education may further increase the disparities. Similarly, individuals who are most likely to lack awareness of their immigration rights and the funds to privately access this information are those with lower education [34]. These individuals are most likely to benefit from public programs such as those that might be offered at the libraries. Individuals diagnosed with or interested in learning more about chronic and mental health illnesses view the libraries as a source of that information [35]. However, the information accessed at the library (e.g., journals, internet searches guided by librarians) may require a high level of literacy and health literacy [36]. Given that literacy and health literacy are correlated with education level [37,38,39], it is imperative that libraries go a step further in information dissemination of health information by offering programs where individuals may learn more about chronic and mental health illnesses. This is especially important for groups with low education who may be hesitant to interact with the health system [33].

## 7. Limitations

A major limitation of this study is that it represents a small sample of the libraries in the US. However, on average, there are 0.22 libraries in a census tract and ~1 library per census tract (in our sample of census tracts) was surveyed, therefore we are confident that our results represent the census tracts surveyed. Despite this limitation, this study provides some helpful insights about library programming in relation to their census tract demographics. It is important to note that the libraries represented in our sample may be biased as directors and librarians who had time and were willing to participate in research may have been more likely to complete our survey. This may also explain our low response rate, another issue limiting the generalizability of our study. Relatedly, the majority of our data was collected prior to the official start of the COVID-19 pandemic in the US. We did not assess for COVID-19 related outcomes, and it is possible that our extremely low response rate at Wave 3 might be explained by the reduced capacity of library staff. Future research should explore how libraries across the U.S. pivoted their programming to meet the needs of their communities during the pandemic and what role they may serve in responding to future pandemics. Although we used census tracts to determine whether the libraries’ programming met the needs of the demographics they serve, libraries may already be meeting the needs of patrons who consistently utilize the library and its programming. However, the limited programming offered by the libraries may contribute to some community members not utilizing the library, thus creating a cycle of exclusion.

## 8. Conclusions

Public libraries have the potential to reduce economic, social, and health inequities in the communities they serve through their programming and practices. Our results suggest that most libraries likely contribute to inequities by offering more programming in communities that may already have the resources for being successful (e.g., more economic mobility programs in higher education communities). These findings highlight gaps in how libraries respond to the needs of their communities through programming. Future research should explore what impacts libraries decision-making for programming and how to improve these decisions to best meet the needs of those who are most likely to experience systemic inequities.

## Figures and Tables

**Table 1:** Descriptives of Proportion of Census Tract Populations in Census Tracts of Surveyed Libraries based on the 2013–2017 American Community Survey 5-year estimates as provided by the National Historical Geographic Information System.

	Mean (SD)	Lowest Value, Highest Value
Demographic Characteristics ^a^		
Highest education ≤ high school diploma/GED *Residents age ≥ 25 with either a GED or High School Diploma or < High School Diploma/Total Population age ≥ 25*	0.41 (0.15)	0.02, 0.74
Non-Hispanic Black *Total non-Hispanic Black residents/Total Population*	0.11 (0.20)	0.00, 1.00
Non-Hispanic NAAN *Total non-Hispanic Native American and Alaskan Native residents/Total Population*	0.02 (0.07)	0.00, 0.82
Non-Hispanic Asian *Total non-Hispanic Asian residents/Total Population*	0.03 (0.05)	0.00, 0.31
Non-Hispanic NHPI *Total non-Hispanic Native Hawaiian and Pacific Islander residents/Total Population*	0.002 (0.007)	0.00, 0.06
Hispanic/Latinx *Total Hispanic or Latinx residents/Total Population*	0.11 (0.15)	0.00, 0.85
Unemployed *Total Population age ≥ 16 years Unemployed in Civilian Labor Force/Total Population age ≥ 16 years in Civilian Labor Force*	0.07 (0.04)	0.00, 0.23
Poverty *Total Population in Poverty/Total Population*	0.16 (0.11)	0.02, 0.51

Notes. GED = General Education Development; NAAN = Native American or Alaska Native, NHPI = Native Hawaiian or Other Pacific Islander; SD = standard deviation. ^a^ Numbers expressed in proportions.

**Table 2:** Descriptives of Programming for Libraries Surveyed (*n* = 235).

	*n* (%)	Mean (SD)
**Economic Mobility Programming**		
How often does your library offer programs/workshops on financial literacy?		2.37 (2.24)
Not offered	107 (45.5)	
Less frequent than once per year	0	
Once per year	0	
A few times per year	7 (3)	
A few times per month	81 (34.5)	
A few times per week	28 (11.9)	
Daily	12 (5.1)	
How often does your library offer programs/workshops on job preparedness?		2.15 (2.05)
Not offered	101 (43)	
Less frequent than once per year	2 (0.9)	
Once per year	16 (6.8)	
A few times per year	30 (12.8)	
A few times per month	60 (25.5)	
A few times per week	14 (6)	
Daily	12 (5.1)	
How often are small business ownership programs/workshops offered?		1.24 (1.94)
Not offered	163 (69.4)	
Less frequent than once per year	0	
Once per year	3 (1.3)	
A few times per year	13 (5.5)	
A few times per month	41 (17.4)	
A few times per week	8 (3.4)	
Daily	7 (3)	
How often are college preparation programs/workshops offered?		1.49 (2.16)
Not offered	155 (66)	
Less frequent than once per year	0	
Once per year	4 (1.7)	
A few times per year	3 (1.3)	
A few times per month	44 (18.7)	
A few times per week	16 (6.8)	
Daily	13 (5.5)	
**Social mobility programming**		
How often are tenant rights, housing, and/or home ownership programs/workshops offered?		1.22 (2.06)
Not offered	172 (73.2)	
Less frequent than once per year	0	
Once per year	0	
A few times per year	2 (0.9)	
A few times per month	35 (14.9)	
A few times per week	16 (6.8)	
Daily	10 (4.3)	
How often are programs/workshops on immigration rights and issues offered?		0.75 (1.75)
Not offered	197 (83.8)	
Less frequent than once per year	0	
Once per year	1 (0.4)	
A few times per year	0	
A few times per month	19 (8.1)	
A few times per week	10 (4.3)	
Daily	8 (3.4)	
How often are English language learning programs/workshops offered?		0.69 (1.31)
Not offered	177 (75.3)	
Less frequent than once per year	3 (1.3)	
Once per year	25 (10.6)	
A few times per year	17 (7.2)	
A few times per month	10 (4.3)	
A few times per week	1 (0.4)	
Daily	2 (0.9)	
How often are programs/workshops on signing up for and/or understanding health insurance?		1.66 (2.21)
Not offered	147 (62.5)	
Less frequent than once per year	0	
Once per year	0	
A few times per year	6 (2.6)	
A few times per month	50 (21.3)	
A few times per week	20 (8.5)	
Daily	12 (5.1)	
**Health-related programming**		
How often are programs/workshops on nutrition and/or healthy eating offered?		2.48 (2.88)
Not offered	133 (56.6)	
Less frequent than once per year	0	
Once per year	1 (0.4)	
A few times per year	1 (0.4)	
A few times per month	7 (3)	
A few times per week	8 (3.4)	
Daily	85 (36.2)	
How often are programs/workshops on physical activity education offered?		1.68 (1.96)
Not offered	128 (54.5)	
Less frequent than once per year	0	
Once per year	14 (6)	
A few times per year	25 (10.6)	
A few times per month	55 (23.4)	
A few times per week	6 (2.6)	
Daily	7 (3)	
How often are programs/workshops on specific chronic illnesses offered?		1.29 (1.99)
Not offered	163 (69.4)	
Less frequent than once per year	0	
Once per year	0	
A few times per year	8 (3.4)	
A few times per month	45 (19.1)	
A few times per week	14 (6)	
Daily	5 (2.1)	
How often are programs/workshops on mental health offered?		1.39 (2.11)
Not offered	161 (68.5)	
Less frequent than once per year	0	
Once per year	0	
A few times per year	8 (3.4)	
A few times per month	38 (16.2)	
A few times per week	18 (7.7)	
Daily	10 (4.3)	

**Table 3:** Results of Bivariate Linear Regressions of the Relationship between Census Tract Demographics and Economic Mobility Programs Offered at Libraries in Census Tracts.

	Bivariate ^a^				Multivariate ^b^			
	Financial Literacy	Job Preparedness	Small Business	College Preparation	Financial Literacy	Job Preparedness	Small Business	College Preparation
	*β* (SE)	*β* (SE)	*β* (SE)	*β* (SE)	*β* (SE)	*β* (SE)	*β* (SE)	*β* (SE)
Highest education ≤ HS diploma/GED	−0.13 (0.98)	−0.03 (0.91)	−0.17 ** (0.85)	−0.26 *** (0.92)	−0.10 (1.28)	−0.09 (1.17)	−0.28 ** (1.07)	−0.22 ** (1.13)
Non-Hispanic Black	0.07 (0.74)	0.17 * (0.67)	0.13 * (0.64)	−0.03 (0.71)	0.10 (0.86)	0.23 ** (0.79)	0.20 ** (0.72)	0.09 (0.77)
Non-Hispanic NAAN	−0.05 (2.00)	−0.01 (1.84)	−0.06 (1.73)	−0.07 (1.93)	−0.08 (2.12)	0.02 (1.94)	−0.06 (1.78)	−0.08 (1.88)
Non-Hispanic Asian	0.14 * (3.04)	1.25 (2.81)	0.17 ** (2.62)	0.36 *** (2.77)	0.13 (3.43)	0.06 (3.15)	0.06 (2.88)	0.27 *** (3.05)
Non-Hispanic NHPI	0.09 (21.80)	−0.02 (20.10)	−0.11 (18.84)	0.10 (21.02)	0.09 (23.64)	−0.02 (21.69)	0.13 (19.84)	0.08 (20.99)
Hispanic/Latinx	−0.11 (0.95)	0.11 (0.87)	0.07 (0.83)	0.16 * (0.91)	−0.89 (1.02)	0.16 * (0.94)	0.11 (0.86)	0.19 ** (0.91)
Unemployed	0.01 (3.72)	0.06 (3.41)	−0.01 (3.22)	−0.10 (3.57)	0.04 (4.53)	0.02 (4.15)	−0.04 (3.80)	−0.03 (4.02)
Poverty	−0.05 (1.41)	0.04 (1.29)	0.07 (1.22)	−0.06 (1.36)	−0.06 (1.86)	−0.06 (1.70)	0.11 (1.56)	−0.05 (1.65)

Notes. ELL = English language learning; GED = General Education Development; HS = high school; NAAN = Native American or Alaska Native, NHPI = Native Hawaiian or Other Pacific Islander. ^a^ R^2^ ranged from <0.01–0.13; ^b^ R^2^ ranged from <0.01–0.21; * *p* < 0.05; ** *p* < 0.01; *** *p* < 0.001.

**Table 4:** Results of Multivariate Linear Regressions of the Relationship between Census Tract Demographics and Social Programs Offered at Libraries in Census Tracts.

	Bivariate ^a^				Multivariate ^b^			
	Health Insurance	Housing	ELL	Immigration Rights	Health Insurance	Housing	ELL	Immigration Rights
	*β* (SE)	β (SE)	*β* (SE)	*β* (SE)	*β* (SE)	*β* (SE)	*β* (SE)	*β* (SE)
Highest education ≤ HS diploma/GED	−0.02 (0.98)	**−0** **.** 17 * (0.90)	−0.10 (0.58)	**−0** .16 * (0.76)	0.03 (1.24)	**−0** .26 ** (1.15)	**−0** .18 * (0.75)	**−0** .28 ** (0.95)
Non-Hispanic Black	0.12 (0.73)	0.10 (0.68)	0.03 (0.43)	0.03 (0.58)	0.14 (0.84)	0.16 * (0.77)	0.03 (0.50)	0.07 (0.64)
Non-Hispanic NAAN	−0.10 (1.96)	−0.05 (1.84)	−0.08 (1.17)	−0.02 (1.56)	−0.15 * (2.07)	−0.08 (1.90)	−0.07 (1.24)	−0.06 (1.58)
Non-Hispanic Asian	0.02 (3.03)	0.13 * (2.80)	0.13 * (1.79)	0.24 *** (2.33)	0.05 (3.35)	0.02 (3.08)	0.08 (2.01)	0.11 (2.55)
Non-Hispanic NHPI	0.10 (21.48)	0.16 * (19.88)	−0.05 (12.82)	0.13 (16.96)	0.14 * (23.05)	0.19 ** (21.24)	−0.06 (13.81)	**0.11 ^a^ (17.59)**
Hispanic/Latinx	−0.10 (0.94)	0.05 (0.88)	0.15 * (0.56)	0.21 ** (0.73)	−0.05 (1.00)	0.11 (0.92)	0.15 * (0.60)	0.23 ** (0.76)
Unemployed	0.07 (3.66)	0.05 (3.41)	0.05 (2.18)	0.08 (2.89)	0.11 (4.41)	0.08 (4.07)	0.10 (2.64)	**0.13 ^a^ (3.37)**
Poverty	−0.10 (1.38)	−0.01 (1.30)	0.06 (0.83)	0.06 (1.10)	**−0** .22 ** (1.81)	−0.02 (1.67)	0.05 (1.08)	0.03 (1.38)

Notes. ELL = English language learning; GED = General Education Development; HS = high school; NAAN = Native American or Alaska Native, NHPI = Native Hawaiian or Other Pacific Islander. ^a^ R^2^ ranged from <0.01–0.06; ^b^ R^2^ ranged from <0.01–0.15; * *p* < 0.05; ** *p* < 0.01; *** *p* < 0.001.

**Table 5:** Results of Bivariate and Multivariate Linear Regressions of the Relationship between Census Tract Demographics and Health-Related Programs Offered at Libraries in Census Tracts.

	Bivariate ^a^				Multivariate ^b^			
	Nutrition	Physical Activity	Chronic Illnesses	Mental Health	Nutrition	Physical Activity	Chronis Illnesses	Mental Health
	*β* (SE)	*β* (SE)	*β* (SE)	*β* (SE)	*β* (SE)	*β* (SE)	*β* (SE)	*β* (SE)
Highest education ≤ HS diploma/GED	0.05 (1.27)	−0.06 (0.87)	**−0** .14 * (0.87)	**−0** .22 ** (0.91)	0.02 (1.66)	−0.06 (1.13)	**−0** .18 * (1.14)	**−0** .29 ** (1.18)
Non-Hispanic Black	−0.11 (0.95)	0.03 (0.65)	0.10 (0.66)	0.04 (0.70)	−0.13 (1.12)	0.04 (.76)	0.17 * (0.77)	0.12 (0.80)
Non-Hispanic NAAN	0.05 (2.57)	−0.01 (1.75)	−0.05 (1.78)	−0.09 (1.88)	0.03 (2.76)	0.05 (1.88)	−0.03 (1.89)	−0.08 (1.96)
Non-Hispanic Asian	**−0** .13 * (3.92)	−0.10 (2.68)	0.13 (2.72)	0.05 (2.89)	**−0** .15 * (4.47)	0.12 (3.04)	0.08 (3.06)	−0.06 (3.18)
Non-Hispanic NHPI	0.02 (28.16)	−0.12 (19.04)	0.04 (19.48)	0.06 (20.57)	0.04 (30.79)	**−0** .16 * (20.93)	0.05 (21.06)	0.13 (21.90)
Hispanic/Latinx	0.03 (1.23)	0.00 (0.84)	−0.004 (0.85)	−0.02 (0.90)	0.03 (1.33)	−0.02 (0.91)	0.03 (0.91)	0.04 (0.95)
Unemployed	−0.02 (4.78)	−0.03 (3.26)	−0.05 (3.31)	−0.12 (3.48)	−0.01 (5.90)	−0.04 (4.01)	−0.08 (4.03)	−0.11 (4.19)
Poverty	−0.01 (1.81)	0.04 (1.23)	0.01 (1.26)	−0.02 (1.33)	0.03 (2.42)	0.08 (1.64)	0.05 (1.65)	0.12 (1.72)

Notes. ELL = English language learning; GED = General Education Development; HS = high school; NAAN = Native American or Alaska Native, NHPI = Native Hawaiian or Other Pacific Islander. ^a^ R^2^ ranged from <0.01–0.05; ^b^ R^2^ ranged from <0.01–0.09. * *p* < 0.05; ** *p* < 0.01.

## Data Availability

Data is available from the corresponding author upon reasonable request.
