# Correction: Fleary et al. Census Tract Demographics Associated with Libraries’ Social, Economic, and Health-Related Programming. *Int. J. Environ. Res. Public Health* 2022, *19*, 6598

**DOI:** 10.3390/ijerph20156471

**Published:** 2023-07-28

**Authors:** Sasha A. Fleary, Carolina Gonçalves, Patrece L. Joseph, Dwayne M. Baker

**Affiliations:** 1Community Health and Social Sciences, CUNY Graduate School of Public Health and Health Policy, New York, NY 10027, USA; 2Eliot-Pearson Department of Child Study and Human Development, Tufts University, Medford, MA 02155, USA; 3Gillings School of Global Public Health, University of North Carolina, Chapel Hill, NC 27514, USA; 4Urban Studies Department, CUNY Queens College, Queens, NY 11367, USA

The authors wish to make the following corrections to this paper [1]:

## Missing Citation

In the original publication, **Marmot (2005)**, **reference 13** was not cited. The citation has now been inserted in ***Section 1***, ***paragraph 3*** and should read:

Systemic inequities rooted in marginalization, racism, and discrimination account for poor health [8,9], social and economic immobility [9,10], and lower quality of life [11] for underserved groups. Improving the wellbeing of impacted communities requires changes to how systems function as well as improved and equitable access to resources. As the role of the public library evolves, it is important to consider how this trusted system may serve to improve the conditions in which people live, work, grow, and learn [12,13], that is, their social determinants of health. Libraries may play a pivotal role in reducing inequities through their programming, especially if they are responsive to the needs of their patrons. For example, libraries in communities with high rates of recently incarcerated or currently incarcerated individuals may provide re-integration and/or family support programming [14]. Such programs have the potential to reduce recidivism and improve economic mobility, and family mental health and well-being—all of which impact long-term health outcomes and quality of life. In other examples, staff at libraries spend a lot of time providing assistance to individuals from vulnerable populations in addition to offering multiple social determinants of health-related programming [5,6].

13. Marmot, M. Social determinants of health inequalities. *Lancet*
**2005**, *365*, 1099–1104.

## Text Correction

There was an error in the original publication. **The name of the institutional review board that oversaw the study was redacted.** A correction has been made to the ***Section 3***, ***paragraph 1***:

The Tufts University Social, Behavioral and Educational Research Institutional Review Board deemed this study as exempt. Librarians from public libraries across the United States were recruited to complete a short survey about the programming offered at their library. Data from the 2017 Public Libraries Survey (*n* = 17,452 library entries) were used to randomly recruit libraries for participation in our study. First data were cleaned to exclude libraries that were open less than 48 weeks per year, only included book mobiles, and were closed or temporarily closed (*n* = 1448 libraries removed). The libraries for each state were sorted into separate files by state and locale. Locales represented the size of the community in which the library was located and its proximity to urban and metropolitan areas. There were four locales: cities, suburbs, towns, and rural areas. All libraries were assigned a unique number (*n* = 16,004 libraries) and a random number generator was used to randomly select 10 libraries from each locale within each state.

There was an error in the original publication. **Reference 36 was incorrectly cited and the word what was removed from a sentence.** A correction has been made to the ***Section 6***, ***paragraph 6***:

The inequities associated with low education and low income are often compounded for individuals who belong to extremely vulnerable groups including those without adequate housing, English language learners, immigrants, and those with chronic and mental illnesses [32]. These groups navigate complicated systems and may therefore be in dire need of resources and guidance for successfully navigating these historically racist and discriminatory spaces [33]. Language barriers may hinder economic mobility, self-advocacy, and health outcomes and these barriers may be most pronounced for individuals with low education. Therefore, fewer English language learner programs in census tracts with lower education may further increase the disparities. Similarly, individuals who are most likely to lack awareness of their immigration rights and the funds to privately access this information are those with lower education [34]. These individuals are most likely to benefit from public programs such as those that might be offered at the libraries. Individuals diagnosed with or interested in learning more about chronic and mental health illnesses view the libraries as a source of that information [35]. However, the information accessed at the library (e.g., journals, internet searches guided by librarians) may require a high level of literacy and health literacy [36]. Given that literacy and health literacy are correlated with education level [37–39], it is imperative that libraries go a step further in information dissemination of health information by offering programs where individuals may learn more about chronic and mental health illnesses. This is especially important for groups with low education who may be hesitant to interact with the health system [33].

## References

With this correction, the order of some references has been adjusted accordingly.

The authors state that the scientific conclusions are unaffected. This correction was
approved by the Academic Editor. The original publication has also been updated.
